# The Association Between the Location of Subarachnoid Hemorrhage and the Occurrence of Takotsubo Cardiomyopathy: A Systematic Review and Meta-analysis

**DOI:** 10.7759/cureus.62533

**Published:** 2024-06-17

**Authors:** Asia Eter, Tomohiro Yamamoto, Aristides Koutrouvelis, Satoshi Yamamoto

**Affiliations:** 1 School of Medicine, University of Texas Medical Branch at Galveston, Galveston, USA; 2 School of Medicine, University of Gunma, Maebashi, JPN; 3 Anesthesiology, University of Texas Medical Branch at Galveston, Galveston, USA

**Keywords:** aneurysm, critical care, stress cardiomyopathy, takotsubo cardiomyopathy, subarachnoid hemorrhage, cerebral circulation

## Abstract

Takotsubo cardiomyopathy (TCM) is a syndrome characterized by transient regional cardiac dysfunction of the left ventricle. The goal of this review is to better understand the relationship between the anatomic locations of subarachnoid hemorrhages (SAHs) and the development of TCM as identified through a review of cohort studies. From inception to December 2023, we systematically explored major electronic medical information sources to identify cases of TCM that developed after SAHs. The six selected studies included in the meta-analysis suggest a modest but statistically significant increase in the odds of the events in the posterior circulation group compared to the anterior circulation group, with a combined odds ratio (OR) estimate of around 1.45-1.46. The fixed effect model gives an overall OR of 1.45 with a 95% confidence interval (CI) of 1.01 to 2.10, z = 2.01, p = 0.0442, while the random effects model yields a slightly higher OR of 1.46 with the same 95% CI, z = 2.03, p = 0.0425. There is a tendency for SAH occurrence in the posterior cerebral circulation to cause SAH-related TCM more frequently than in the anterior cerebral circulation.

## Introduction and background

Takotsubo cardiomyopathy (TCM), also known as stress-induced cardiomyopathy or transient apical ballooning syndrome, has emerged as a topic of significant interest in recent years due to its potential association with subarachnoid hemorrhage (SAH). While the general population's SAH incidence has decreased over past decades, possibly in part related to lifestyle changes [1], it remains a recognized trigger for TCM development. Importantly, TCM affects a relatively small proportion of hospitalized patients, with estimates suggesting a prevalence of approximately 0.02% in the United States [2].

TCM is characterized by a transient left ventricular dysfunction in the absence of obstructive coronary artery disease. Acute medical or stressful events, encompassing both physical and emotional stressors, are known triggers for this condition [3]. Due to overlapping clinical presentations, TCM can be misdiagnosed as acute coronary syndrome (ACS), particularly following SAH. Research suggests a misdiagnosis rate of 1-2% in patients suspected of having ACS, highlighting the importance of accurate diagnosis for TCM [4-6]. Furthermore, similar to ACS, TCM carries a risk of cardiogenic shock in roughly 10% of cases, underlining the potential for severe complications [7].

In the context of SAH management, identifying patient subgroups at high risk for severe complications is crucial. Therefore, recognizing TCM as a potential complication of SAH holds significant clinical implications. Heightened awareness among healthcare providers managing patients with neurological emergencies is essential. Early recognition and management of TCM in the setting of SAH are paramount, as prompt intervention may improve patient outcomes and mitigate adverse events such as heart failure and mortality.

SAH is a critical event identified by bleeding into the subarachnoid space, frequently attributed to the rupture of intracranial aneurysms, which account for about 85% of instances [8]. The clinical presentation of SAH varies depending on the location of the ruptured aneurysm. The cerebral blood supply is intricately divided into two main systems: the anterior circulation (AC) and the posterior circulation (PC); the AC supplies blood to the frontal and temporal lobes of the brain, and the PC supplies blood to the occipital and parietal lobes, brainstem, and cerebellum [9]. The diffuse or anterior location of the aneurysm suggests a higher risk of rebleeding, necessitating close monitoring and potentially more aggressive treatment strategies [10]. Dissecting aneurysms and those located in the PC have distinct presentations, such as luminal narrowing of blood vessels and oculomotor nerve palsies affecting eye movement, respectively [11]. While the reported occurrences of TCM after SAH vary, no research has investigated the association between the specific anatomical locations of ruptured aneurysms and the development of TCM in relation to cerebral blood flow patterns. The relationship between the anatomical locations of ruptured cerebral aneurysms and TCM occurrence remains unclear. This knowledge gap represents a crucial area for further investigation.

This systematic review aims to explore current cohort study literature to examine this potential association. By systematically analyzing available data, we seek to understand the complexities surrounding the SAH-TCM link. Specifically, we aim to identify potential risk factors associated with specific aneurysm locations and their influence on TCM development. Furthermore, this review will propose avenues for future research to elucidate the underlying mechanisms linking the anatomical locations of aneurysms and TCM susceptibility.

## Review

Methods

Search Strategy

We systematically explored major electronic medical information sources, including PubMed, MEDLINE, Google Scholar, ScienceDirect, BMJ databases, and Cochrane databases to identify cohort studies from inception to December 2023 of TCM or stress cardiomyopathy with SAH in cooperation with a professional librarian. To ensure that no additional articles were missed, reference lists of the retrieved articles were reviewed. Two authors (AE and TY) independently extracted the data. Discrepancies were resolved by consensus among the authors. After the search, two independent reviewers selected the articles that met the inclusion criteria, had human subjects, and were in English. This review was registered with the International Platform of Registered Systematic Review and Meta-analysis Protocols (INPLASY) with registration number INPLASY202410057.

Study Selection

Studies that described patients with stress cardiomyopathy or TCM in the setting of SAH were included. Classical stress cardiomyopathy was defined as apical ballooning, apical hypokinesis, or apical akinesis. The included studies had to explicitly mention stress cardiomyopathy or TCM as the target patient population with the exclusion of studies conducted solely on stress cardiomyopathy variants or neuro-cardiogenic injury patients. Case reports, case series, traumatic SAH, and patients with cerebral hemorrhage other than SAH were excluded.

Patient Population, Data Collection

For statistical analysis, demographic and clinical characteristics of patients from published series and patients with SAH-related TCM were compared by summary odds ratios (ORs) constructed using both the fixed effects Mantel-Haenszel method and random effects DerSimonian and Laird method. The p-values were two-tailed, with statistical significance set at 0.05, and the confidence interval (CI) was calculated at a 95% level for all statistical analyses. The meta-analysis was conducted using R statistics (R Foundation for Statistical Computing, Vienna, Austria).

The size of the effect for quantitative outcomes was extracted from the mean and standard deviation plus the population size for each study. It was calculated as a difference in means (DM) with a CI and p-value. The neutral DM was 0, and the 95% CI crossing 0 was considered not significant; p values were significant if < 0.05. The size of the effect for dichotomous outcomes was calculated from the incidence rate and size of the groups and expressed as odds ratio (OR), 95% CI, and p-value. An OR of 1 represented a neutral effect for the outcome. A CI crossing 0 was considered not significant, as well as p values above 0.05. The choice of fixed vs. random effects analysis was decided in favor of the latter because of the heterogeneity of the actions. When the number of studies with usable data for a given outcome is a few, a fixed effect analysis is performed because of the uncertainty between the studies' variances. A heterogeneity test was performed including the comparison of Cochrane's Q with the degrees of freedom (df), the variance of true effects (T2), and the real difference in effect size (I2), but this information was not used to decide the type of analysis (fixed vs. random) to be used. R statics were used for analysis. This report was written following the Newcastle-Ottawa scale [12,13].

Results

**Figure 1:**
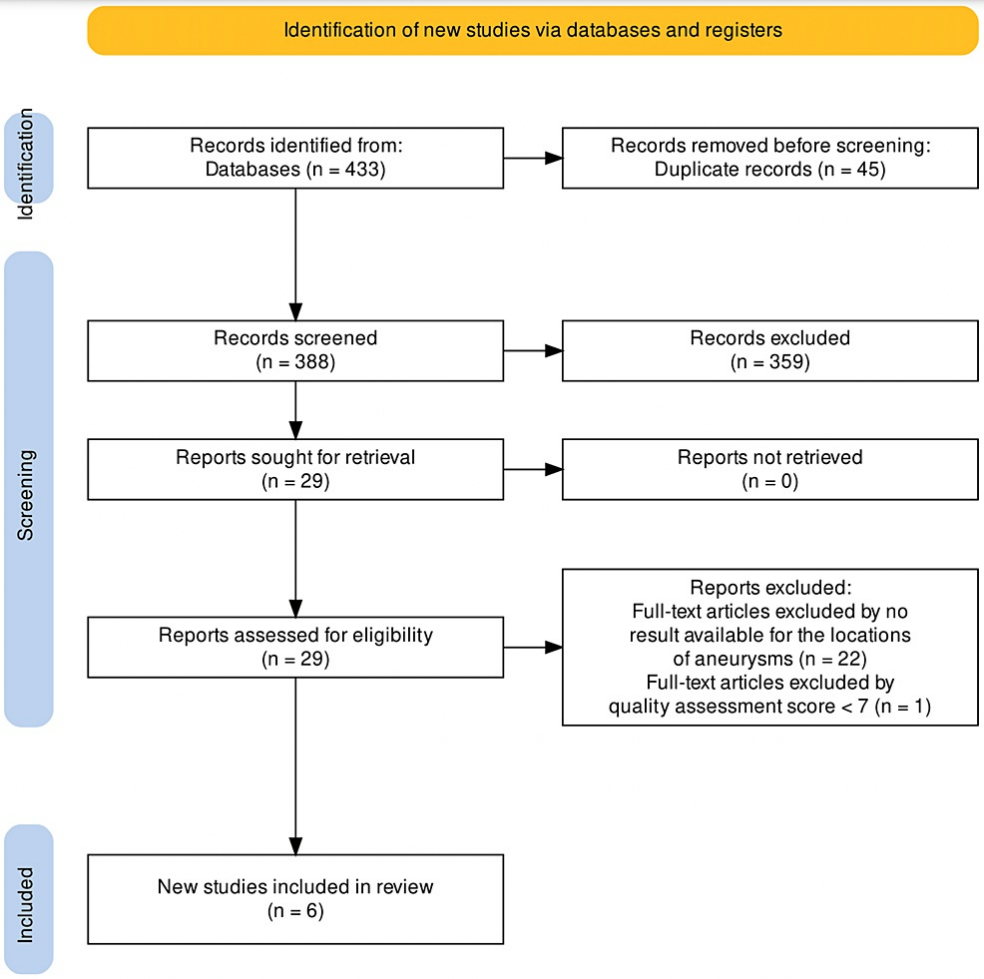
PRISMA flow diagram of the study selection process PRISMA: Preferred Reporting Items for Systematic Reviews and Meta-Analyses

The literature search rendered 433 studies. After removing duplicates, 45 studies were ruled out and 359 studies were screened out by title and abstract reviews. Out of the 29 retrieved, 22 full-text articles were excluded by no result available for the location of the aneurysm. One article was excluded based on a quality assessment (less than a score of seven) by the Newcastle-Ottawa scale. Six was the final number of studies considered [10,14-18]. This systematic review was conducted following the Preferred Reporting Items for Systematic Reviews and Meta-Analyses (PRISMA) guidelines and the search flowchart is represented in Figure 1.

**Figure 2:**
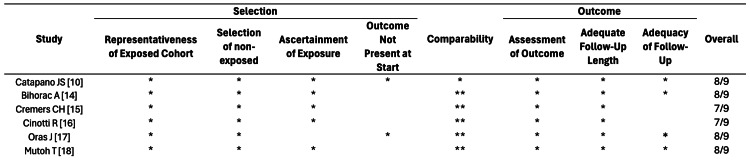
Newcastle-Ottawa Scale for risk of quality assessment of the included studies (scores ≥ 7–9, 4–6, <4 are considered low, intermediate, and high risk, respectively) * Equals one point, with a total of nine points (* for each item and ** equals two points for comparability)

The overall quality of studies ranged from 7 to 8 on the Newcastle-Ottawa scale, with a mean value of 7.7, indicating a low-risk quality. Figure 2 illustrates the quality of studies included in the systematic review based on the Newcastle-Ottawa scale.

**Figure 3:**
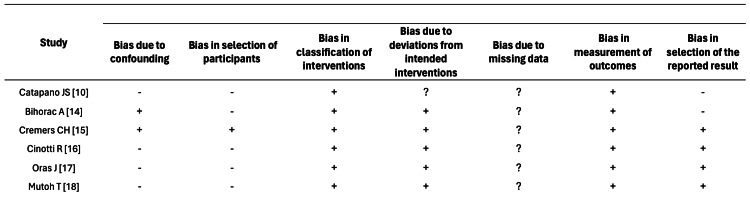
Risk of bias summary Review of authors’ judgment about each risk of bias item using Cochrane Collaboration’s Risk Assessment Tool for each included trial + indicates low risk of bias; -, high risk of bias; ?, unclear risk of bias

The overall risk of bias for the included studies was measured using the Cochrane Collaboration’s Risk Assessment Tool. The available evidence is summarized in a readable form in Figure 3.

The summary of characteristics of each cohort study, including age, gender, and various anatomic locations of SAH that led to the development of TCM can be found in Table 1 and those that did not lead to the development of TCM can be found in Table 2. Among the patients included in the six studies who developed TCM after SAH, 78% were female with a median age of 60 years. In contrast, of patients who experienced SAH but did not develop TCM, 59% were female with a median age of 57.55 years. Additionally, 60% of patients who developed TCM had SAH in the anterior cerebral circulation, while 40% had SAH in the posterior cerebral circulation. Patients who experienced an SAH but did not develop TCM had a slightly higher proportion of SAH in the anterior cerebral circulation at 61%, compared to 37% in the posterior cerebral circulation. 

**Table 1:** Characteristics of studies with subarachnoid hemorrhages and subsequent takotsubo cardiomyopathy development AC: anterior circulation; PC: posterior circulation; SAH: subarachnoid hemorrhage

						Location of SAH			
Study	Total number of patients	Total number of male patients	Number of female patients (N)	Number of female patients (%)	Mean age in years	AC (N)	AC (%)	PC (N)	PC (%)
Catapano JS [10]	25	1	24	2%	54.5	12	1%	13	1%
Bihorac A [14]	54	8	46	25%	59	28	15%	26	14%
Cremers CH [15]	35	6	29	40%	61.9	25	35%	10	14%
Cinotti R [16]	15	6	9	22%	53	11	27%	4	10%
Oras J [17]	36	14	22	13%	61	26	16%	10	6%
Mutoh T [18]	20	6	14	30%	65	9	20%	11	24%

**Table 2:** Characteristics of studies with SAHs and no subsequent takotsubo cardiomyopathy development AC: anterior circulation; PC: posterior circulation; SAH: subarachnoid hemorrhage

						Location of SAH			
Study	Total number of patients	Total number of male patients	Number of female patients (N)	Number of female patients (%)	Mean age in years	AC (N)	AC (%)	PC (N)	PC (%)
Catapano JS [10]	978	395	583	58%	55.3	555	55%	398	40%
Bihorac A [14]	129	36	93	51%	56	80	44%	49	27%
Cremers CH [15]	37	9	28	39%	58.1	27	38%	10	14%
Cinotti R [16]	26	11	15	37%	57	20	49%	6	15%
Oras J [17]	127	82	45	28%	60	108	66%	19	12%
Mutoh T [18]	26	8	18	39%	67	14	30%	12	26%

**Figure 4:**
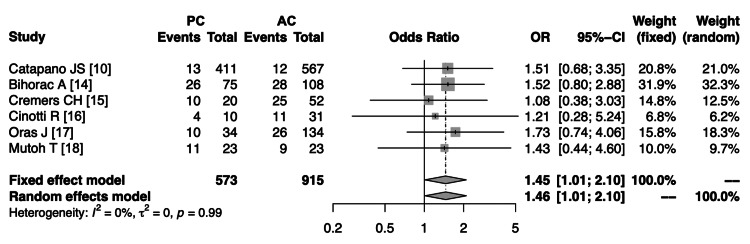
Summary of fixed and random effects for takotsubo cardiomyopathy with PC vs. AC subarachnoid hemorrhages AC: anterior circulation; PC: posterior circulation; CI: confidence interval; OR: odds ratio

The summary of fixed and random effects for TCM with PC vs. AC SAH is shown in Figure 4. The fixed effect model gave an overall OR of 1.45 with a 95% CI of 1.01 to 2.10, z = 2.01, p = 0.0442, while the random effects model yielded a slightly higher OR of 1.46 with the same 95% CI, z = 2.03, p = 0.0425. The studies appeared to be fairly consistent, with most individual ORs and their confidence intervals falling on the side favoring the experimental group (OR > 1). However, each of the studies had a wide CI crossing 1, indicating no significant difference between groups. The heterogeneity measures (I2 = 0%, τ2 = 0, p = 0.99) suggested a low heterogeneity among the included studies, which supports the use of the fixed effect model (Q = 0.57, df = 5). Overall, the meta-analysis suggested a modest but statistically significant increase in the odds of the event in the PC group compared to the AC group, with a combined OR estimate of around 1.45-1.46.

Discussion

TCM is a serious complication of subarachnoid hemorrhage. The development of TCM following SAH presents a complex interaction between neurological and cardiac pathologies. Although previously considered a relatively benign and reversible condition, recent studies have shown TCM to be associated with significant morbidity and mortality [19-21]. In our systematic review, we confirm that there is a tendency for ruptured aneurysms located in the PC to cause SAH-related TCM more frequently than in the AC. It is noteworthy to add that the PC contributes approximately 18-24% to the total global cerebral blood flow [22]. Further, aneurysms in the PC are less common, accounting for 4-4.9% of clinical presentations [23]. Interestingly, patients who developed TCM had a higher neurological severity grading on presentation [20]. According to Cremers et al., they found that prolonged cardiac dysfunction increased the risk of delayed cerebral ischemia [15]. Mutoh et al. reported poor three-month functional outcomes in postoperative SAH patients with TCM [18]. These findings suggest the need for a comprehensive cardiovascular evaluation of high-risk SAH patients at the outset to identify secondary TCM, which may indicate a worse prognosis and necessitate transfer to a higher level of care.

Currently, anesthesia management and intensive care for patients with TCM is similar to acute myocardial infarctions, given the similarity in presentation, with the use of heparin, aspirin, and antiplatelet therapy [24]. In the event of surgery or radiologic intervention, the stress contributing to hemodynamic instability associated with placing invasive monitoring procedures before the induction of anesthesia and sedation should be minimized by using local anesthesia, analgesics, and prophylactic beta-blocker therapy [25]. On top of the chosen anesthetic or sedative strategy, it is essential to minimize stressors that might induce an influx of catecholamines. This can be achieved through ensuring smooth transitions during induction and emergence from anesthesia, and appropriate consistent depth of sedation in the ICU [25]. 

One explanation for the association between SAH in the PC and TCM lies in the distribution and functional significance of the affected brain regions. The brain stem is indeed associated with catecholamine release and plays a significant role in the synthesis and regulation of these neurotransmitters, which are vital for various physiological and behavioral processes [26]. A disruption of blood flow in these regions due to SAH-induced vasospasm or ischemia may trigger a dysfunctional sympathetic response, thereby contributing to the pathogenesis of TCM. Notably, brain stem neurons, including those in the human fetus, contain tyrosine hydroxylase, which is crucial for catecholamine synthesis, indicating the presence of catecholamine-producing cells in this region [27]. Several features of TCM suggest that this disorder may be caused by diffuse catecholamine-induced microvascular spasm or dysfunction, resulting in myocardial stunning [28] or by direct catecholamine-associated myocardial toxicity [29]. Moussouttas et al. found an association between elevated plasma norepinephrine and TCM in SAH, suggesting a predominantly neurogenic process mediated by neuronal norepinephrine rather than adrenal epinephrine [30]. These mechanisms help explain the increased probability of SAH in the PC causing TCM compared to the AC, given the differences in sympathetic innervation.

Our results are consistent with the present literature in that post-menopausal women have a higher predilection for the development of TCM after SAH. Moreover, Catapano et al. found that TCM following SAH is more likely to occur in female patients with larger-sized aneurysms [10]. Given these results, a lack of estrogen has been considered a significant risk factor. Estradiol production induces the production of heat shock protein and atrial natriuretic peptide, both of which are thought to be cardioprotective against the adverse effects of catecholamine surge and oxidative stress [31]. Additionally, postmenopausal women might be more susceptible to TCM due to alterations in functional cerebral asymmetry and sympathovagal balance caused by diminishing sex steroids [29].

Although the study offers valuable insights, it is important to acknowledge its limitations. The exclusion of case reports and case series may have limited the data pool regarding the association between SAH location and TCM development. Despite the overall high quality of the evidence presented in the selected articles according to the Newcastle-Ottawa quality scale, it is important to note that all articles are based on observational studies with a lack of randomized controlled trials. Consequently, it may be deemed premature to draw a definitive conclusion at this stage. Therefore, it is imperative that larger, prospective, high-quality studies be conducted to validate the results obtained.

In summary, it is crucial for anesthesiologists and intensivists to be aware of the anatomic propensity of PC SAH to induce TCM when managing these patients. These considerations might be helpful for prompt strategic hemodynamic management and overall improved prognoses for patients with TCM following SAH.

## Conclusions

In conclusion, our findings indicate a higher tendency of TCM to develop after SAH in cases involving the posterior cerebral circulation, particularly in female patients. Early recognition and management of TCM in SAH patients is crucial for improving outcomes with concurrent vigilant monitoring and thorough cardiovascular evaluation. Given some study limitations, this review emphasizes the importance of further research to confirm these findings and improve patient care strategies.
